# Temporal genomic evolution of bird sex chromosomes

**DOI:** 10.1186/s12862-014-0250-8

**Published:** 2014-12-12

**Authors:** Zongji Wang, Jilin Zhang, Wei Yang, Na An, Pei Zhang, Guojie Zhang, Qi Zhou

**Affiliations:** School of Bioscience and Bioengineering, South China University of Technology, Guangzhou, 510006 China; China National GeneBank, BGI-Shenzhen, Shenzhen, 518083 China; Department of Biology, Centre for Social Evolution, University of Copenhagen, Universitetsparken 15, DK-2100 Copenhagen, Denmark; Department of Integrative Biology, University of California, Berkeley, CA94720 USA

**Keywords:** Avian genome, Evolutionary strata, Male-driven evolution, Fast-Z evolution, Dosage compensation

## Abstract

**Background:**

Sex chromosomes exhibit many unusual patterns in sequence and gene expression relative to autosomes. Birds have evolved a female heterogametic sex system (male ZZ, female ZW), through stepwise suppression of recombination between chrZ and chrW. To address the broad patterns and complex driving forces of Z chromosome evolution, we analyze here 45 newly available bird genomes and four species’ transcriptomes, over their course of recombination loss between the sex chromosomes.

**Results:**

We show Z chromosomes in general have a significantly higher substitution rate in introns and synonymous protein-coding sites than autosomes, driven by the male-to-female mutation bias (‘male-driven evolution’ effect). Our genome-wide estimate reveals that the degree of such a bias ranges from 1.6 to 3.8 among different species. G + C content of third codon positions exhibits the same trend of gradual changes with that of introns, between chrZ and autosomes or regions with increasing ages of becoming Z-linked, therefore codon usage bias in birds is probably driven by the mutational bias. On the other hand, Z chromosomes also evolve significantly faster at nonsynonymous sites relative to autosomes (‘fast-Z’ evolution). And species with a lower level of intronic heterozygosities tend to evolve even faster on the Z chromosome. Further analysis of fast-evolving genes’ enriched functional categories and sex-biased expression patterns support that, fast-Z evolution in birds is mainly driven by genetic drift. Finally, we show in species except for chicken, gene expression becomes more male-biased within Z-linked regions that have became hemizygous in females for a longer time, suggesting a lack of global dosage compensation in birds, and the reported regional dosage compensation in chicken has only evolved very recently.

**Conclusions:**

In conclusion, we uncover that the sequence and expression patterns of Z chromosome genes covary with their ages of becoming Z-linked. In contrast to the mammalian X chromosomes, such patterns are mainly driven by mutational bias and genetic drift in birds, due to the opposite sex-biased inheritance of Z vs. X.

**Electronic supplementary material:**

The online version of this article (doi:10.1186/s12862-014-0250-8) contains supplementary material, which is available to authorized users.

## Background

Sex chromosomes of birds and mammals originated independently from different pairs of ancestral autosomes [1,2], and have experienced stepwise suppressions of recombination in parallel [3,4]. Since recombination was lost at different time points, each affected region shows a distinctive level of sequence divergence between sex chromosomes from adjacent regions, manifested as a pattern of ‘evolutionary strata’ [5-9]. As a result of recombination loss, mammalian Y and avian W chromosomes are specific to one sex and usually have lost most functional genes, rendering their counterpart (X and Z chromosomes) hemizygous and asymmetrically transmitted between sexes. These features subject the sex chromosomes to special mutational and selective regimes apart from autosomes, and may select for evolution of dosage compensation on X/Z in response to the unbalanced gene dosages between sex chromosomes and autosomes. Indeed, X chromosomes have evolved numerous unusual properties in their sequence substitution and gene expression relative to autosomes [10,11]. Previous genome-wide studies in mammals consistently found that the X chromosome has a lower level of interspecific sequence divergence at neutral sites relative to autosomes [12,13], due to the effect of ‘male-driven’ evolution. This refers to the fact that males usually undergo many more rounds of germ line cell divisions, thus accumulate more mutations per time unit than females in most animals [14]. The X chromosome spends less time in males, therefore it is expected to have a lower mutation rate than autosomes and Y chromosome. On the other hand, it also tends to experience more effective adaptive evolution and show a faster evolution rate, since its hemizygous state in male would more readily expose the male recessive beneficial mutations to positive selection (‘fast-X’ evolution) [15,16]. Indeed, an accelerated rate of protein sequence evolution has been observed on human and chimpanzee X chromosomes [17,18], and sperm proteins show an even more pronounced pattern of ‘fast-X’ evolution when being X-linked, suggesting they are under strong positive selection [19,20]. Finally, the gene content of X chromosomes of both Drosophila and mammals has become ‘demasculinized’ (deficient for testis genes) or ‘feminized’ (enriched for ovary genes) [21,22]. Sexual antagonism seems to be a direct cause, given that the X chromosome spends more time in females and is expected to accumulate mutations favorable to females at the expense of males [23]. However, later studies challenged the necessity of invoking such an explanation, and showed meiotic sex chromosome inactivation (MSCI) or dosage compensation is sufficient to explain the demasculinized X-linked gene content in mammal [24] or Drosophila [25,26].

These complex forces acting on the X chromosome are also expected to shape the evolution of Z chromosome, but with very different outcomes. Opposite to the X chromosome, the Z chromosome is more often transmitted in males than in females. And recent studies found that the chicken Z does not seem to undergo MSCI and only has evolved regional instead of chromosome-wide dosage compensation like mammalian X chromosomes do [27-29]. These features are likely to produce a distinctive sequence and expression pattern of Z chromosome comparing to that of X chromosome and autosomes. Although ZW sex systems are widely distributed in birds, reptiles, fishes and butterflies, most of our current knowledge on evolution of Z chromosome comes from studies of chicken, which is the first species having genome sequenced among the ZW species. Previous analyses of some intronic fragments show chicken and other birds have a significantly higher level of neutral sequence divergence on Z chromosome than autosomes, indicating a higher Z-linked mutation rate caused by the male-driven evolution effect. This leads to an estimate of male-to-female mutation rate ratio (*α*) ranging from 1.7 to 6.5 in birds [30,31]. Chicken Z-linked genes have also been found to evolve significantly faster in their protein coding sequences than the autosomal genes (‘fast-Z’ effect) [32]. In contrast to the pattern of ‘fast-X’ evolution, Z-linked chicken female-biased or non-biased genes do not show a more pronounced pattern of fast evolution than male-biased genes, suggesting a non-adaptive underlying driving force [33]. Theoretical simulations suggest demographic and mating system factors would impact the X/Z chromosomes more than autosomes in their effective population size (*N*_e_) [15,34,35]. The relative *N*_e_ of Z-linked genes vs. autosomal genes can be further decreased from the neutral expectation of 3/4 due to for example, widespread sexual selection acting on male birds. Such an effect of genetic drift can also produce a pattern of ‘fast-Z’ evolution, through fixation of excessive slightly deleterious mutations on the Z chromosome. This alternative explanation remains to be tested with more empirical data, with multiple species of varying relative *N*_e_ values of Z chromosome, so that to establish its generality. In addition, a general comparison between Z chromosomes vs. autosomes would not only offer a powerful independent test on all the hypotheses proposed for X chromosome evolution, but also provide important insights into the distinctive evolution mode of Z chromosome under different inheritance and gene regulation programs [11].

We have recently reconstructed the evolutionary history of bird Z/W sex chromosomes, based on analyses of a total of 17 species’ genomes [4]. To our surprise, many species besides the known cases of Palaeognathae birds (ostrich and tinamou) [36], degenerate very slowly in their W chromosomes. This is in great contrast to the mammalian Ys or chicken W with few functional genes left [3,37], whose boundaries of evolutionary strata have never been determined due to the scarcity of Y/W linked genes and sequences. We are allowed to infer the ancestral karyotype of bird sex chromosomes with the newly available ostrich genome, and precisely demarcate the evolutionary strata along the Z chromosome of chicken as well as other bird species [4]. Within each stratum, genes have been truly Z-linked for a different time span. This provides a unique system for us to investigate the temporal evolution of sex chromosome for the first time. In this work, we greatly expand our analyses to cover the high-quality genomes of a total of 45 bird species [38], and transcriptomes of 4 bird species that have become recently available [28,39-41]. Comparing to the previous analyses in chicken [32,33,42], the studied species span the entire avian phylogeny, representing the most comprehensive analyses of bird sex chromosomes up to date. With these species’ recently resolved phylogeny based on the genomic dataset [43], we aim to address the following questions: first, how are the sequence and gene expression patterns different between the Z chromosome and autosomes and what are the main causes of such differences? Second, what is the temporal dynamics of such differences accompanied by the stepwise recombination loss between avian sex chromosomes? Finally, there have been continuous efforts of quantifying the life history traits of birds ever since the times of Darwin, which show tremendous variations between species, and frequently between sexes of the same species. The abundant genome resources available for so many bird species provide us great opportunities to associate their trait variations to the patterns of genome evolution, so that to illuminate the possible interaction between the two.

## Results and discussion

### Bird species show different degrees of male-driven evolution

**Figure 1:**
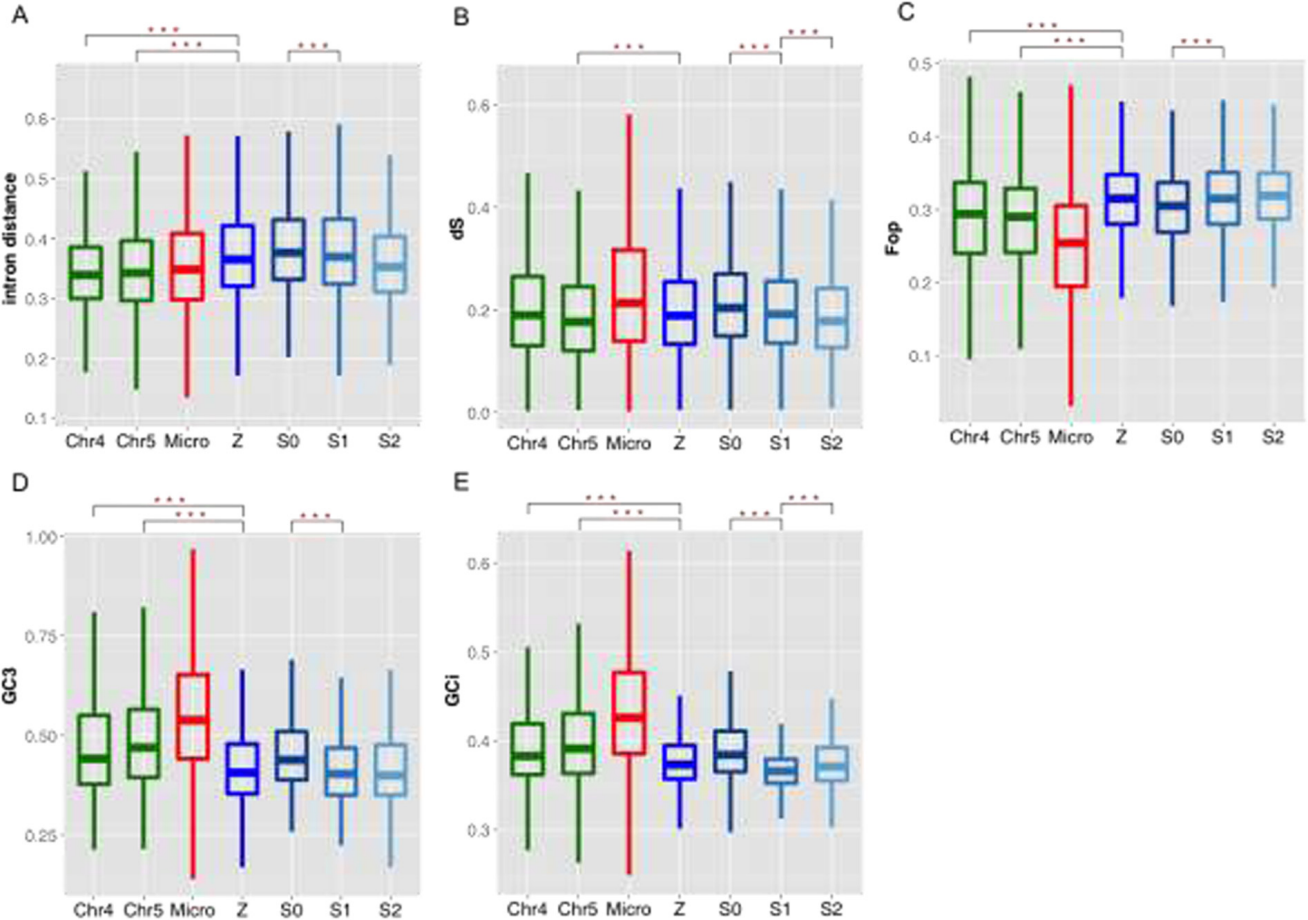
**Evolution patterns of synonymous sites and non-coding regions in bird genomes. (A-E)** We show sequence patterns in introns and synonymous sites of coding regions divided in different chromosome sets or different evolutionary strata (from young to old strata: S2, S1, S0) of Z chromosomes. Microchromosome genes (shown in red) exhibit a very different sequence pattern (*d*S, codon usage bias, G + C content) relative to macro-chromosomes including the Z, due to their different chromosome size and gene densities [50]. Therefore, we mainly compare the patterns of Z-linked genes to those of chr4 and chr5, which have a similar size and gene number. We also performed comparisons between Z chromosome vs. all macro-chromosomes, and the results are similar (Additional file 3: Table S2). We show the Wilcoxon test significance level of differences with asterisks. ^***^:*P*-value < 0.001. *d*S: lineage specific synonymous substitution rate. Fop: frequency of optimal codons. GC3: G + C content at the third codon position. GCi: G + C content of introns.

**Figure 2:**
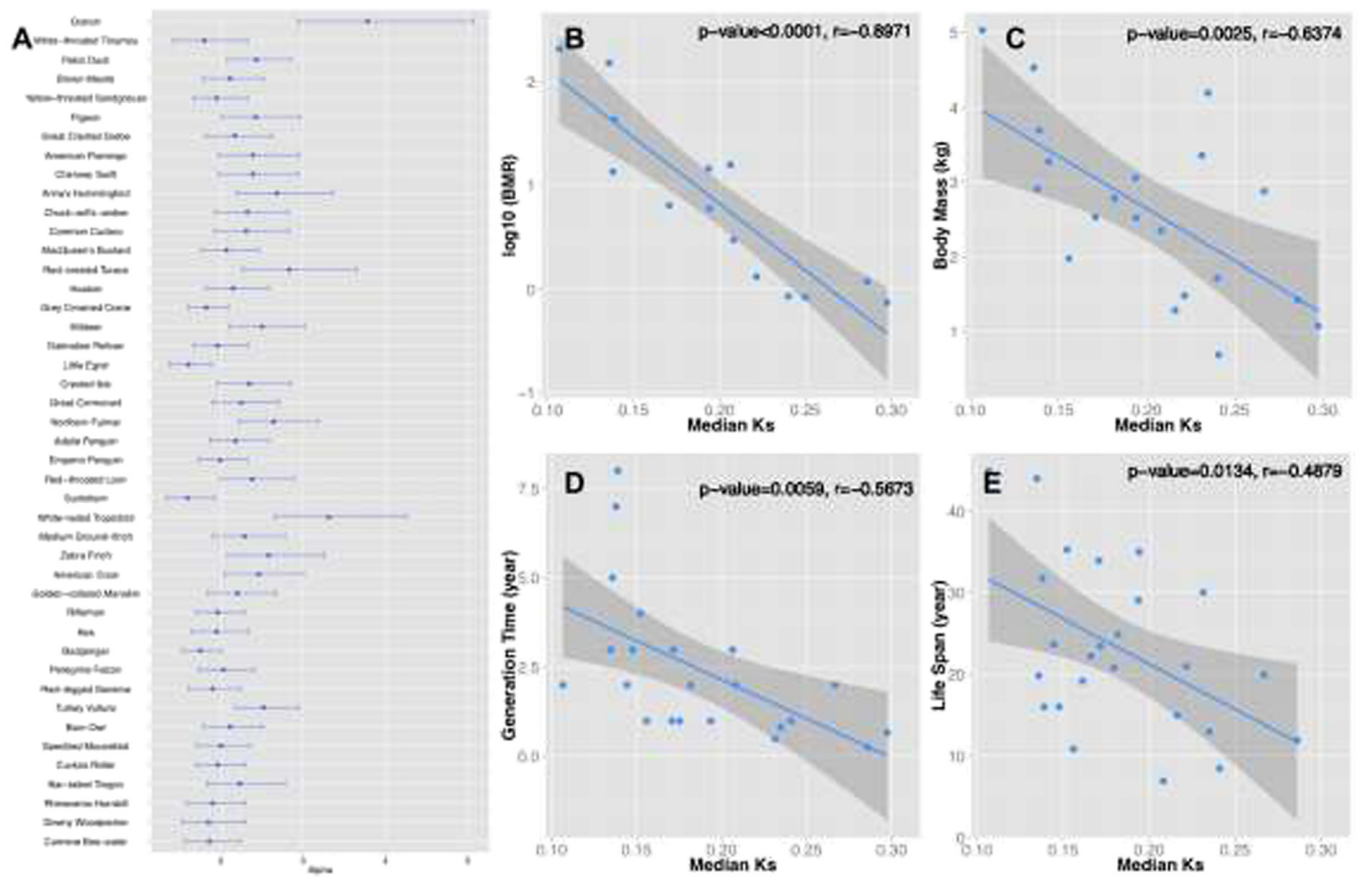
**Male-drive evolution of genomic sequences in birds. (A)**. Male-to-female mutation bias of 45 bird species. We show the degree of male-driven evolution (*α*) inferred from comparing intronic substitution rates between Z chromosomes and macrochromosomes, as well as their confidence intervals. **(B-E)**. The association between synonymous substitution rates vs. life history traits. BMR: Basal Metabolic Rate. The numbers of data points vary by the species with certain life history trait data available.

Previous comparative karyotyping of multiple species has revealed strong conservation of gene content between avian Z chromosomes [44], and also a remarkably low level of inter-chromosomal rearrangements between macro- and micro- chromosomes [45-47], except in a few species [48,49]. This allows us to directly study sequence and expression patterns of genes of other bird species based on their orthologous relationship with chicken. To compare the mutation rates between different chromosomes, we collect orthologous sequence alignments of intronic regions from macro-chromosomes and Z chromosomes. We focus all our analyses throughout this study between these two sets of chromosomes of a similar size, because microchromosomes have very different genomic features (i.e., recombination rate, gene density, GC content, repeat content etc.) compared to others [50], which influence their mutation and selection patterns (also see below). Recent study has identified only about 2% of intronic sequences that are highly conserved across all these species and potentially functional [38]. Therefore introns are expected to evolve predominantly under neutrality, and their difference of nucleotide substitution rates between autosomes vs. Z chromosome, if any would be mainly driven by male mutational bias. In total, we obtain on average 21.8Mb autosomal (i.e., macro-chromosomal) sequences and 465.9 kb Z chromosome sequences per species of 45 species and chicken. Indeed, we find the Z-linked introns show a significantly higher substitution rate (*P* < 2.2e-16, Wilcoxon test) than autosomal introns, and older evolutionary strata (we named the oldest strata as S0, then S1, S2 etc.) show an even higher substitution rate (Figure 1A, Additional file 1: Figure S1). By comparing the median substitution rates between Z and all macrochromosomes [51] (see Methods), we estimate that the degree of male mutation bias *α* ranges from 1.60 (95% confidence interval 1.34 ~ 1.92) in Sunbittern to 3.78 (2.94 ~ 5.07) in Ostrich (Figure 2A, Additional file 2: Table S1), confirming a widespread effect of male-driven evolution among avian lineages. These genome-wide estimates are within the range of previous estimates (1.7 ~ 6.5) [30,31,52,53] using sampled gene pairs or intronic fragments, but with much smaller confidence intervals. Since the amount of data used is the largest for this kind of study in birds, the estimates should be much more accurate and immune to the regional variations of mutation rate.

Many factors affecting numbers of germ line cell divisions or DNA damage may underlie the variation of spontaneous mutation rate and *α* among species. Their associations have been extensively studied in plants [54] and mammals [55,56], but not in birds at a genome-wide level. We further examine a diverse array of life-history traits of the studied 45 species, and their association with lineage-specific synonymous substitution rates (*d*S) and *α*. We find that the median value of lineage specific synonymous substitution rates of autosomal genes, as an approximation of species’ mutation rate, exhibits a significant (*P* < 0.05, Pearson’s correlation test and Chi-square test) negative correlation with species’ life span, generation time, body mass and basal metabolic rate (Figure 2B-E), after controlling for the phylogenetic dependence. This pattern is consistent with the previous findings in mammals [55,56]. Interestingly, we haven’t found a significant correlation between these traits vs. *α,* as a recent study did analyzing 32 mammalian genomes [55] (Additional file 1: Figure S2)*.* This could result from the high variation of both spermatogenesis and oogenesis processes among different bird species (e.g., seasonal vs. continual breeders) [57], which may respectively influence the male and female mutation rates toward different directions.

### Synonymous sites evolve faster in the older strata of Z chromosome

Sex chromosomes and autosomes differ not only in their spontaneous mutation rate, but also in their rate of evolution within coding regions, which directly reflect the different intensities of selection and genetic drift. We further study 306 Z-linked and 5280 macro-chromosome orthologous gene pairs’ coding sequences, which together comprise about 40% of the entire avian gene repertoire. We perform the comparison between the these two chromosome sets (Additional file 3: Table S2), as well as between chrZ vs. chr5 and chr4, to further control for the variation of chromosomal size and gene density within macrochromosomes [58]. If the effect of codon usage bias (CUB) is weak, synonymous sites are expected to evolve nearly neutrally, thus show a pattern of higher substitution rate on the Z-linked loci than autosomal loci similarly as at intronic sites. We found a significant difference of synonymous substitutions rates (*d*S) between the Z-linked and autosomal loci in only 11 out of 45 species (*P <* 0.05, Wilcoxon test, Additional file 3: Table S2), and the pattern of significance depends on the macrochromosome chosen (Figure 1B), implying some degree of natural selection or mutational bias is impacting these species’ synonymous sites. We also find loci that have become Z-linked for a longer time (e.g., genes of S0) tend to have a higher *d*S value (Figure 1B) and this trend is consistent across all the studied species (Additional file 1: Figure S3). Further inspection of different measurement of CUB, including Frequency of Optimal Codons (FOP) (Figure 1C) and codon bias index (CBI) consistently confirms Z-linked genes have a significantly higher degree of CUB (*P* < 0.05, Wilcoxon test) compared to the genes on macro-chromosomes, in almost all the species studied (Additional file 1: Figure S4, Additional file 3: Table S2), and genes that have recently become Z-linked tend to have a higher level of CUB (Figure 1C). Ostrich is the only exception, because 2/3^rd^ of its Z chromosome is still recombining with the W chromosome as a pseudoautosomal region and evolves like an autosome [36,59].

This pattern is in accordance with a higher CUB found on the X chromosomes of *C. elegans*, *Drosophila* and human [13,60], and may reflect a more efficient purifying selection on the hemizygous X- or Z-linked loci, where recessive deleterious mutations are more readily exposed to natural selection [60]. However, genetic drift and mutational bias probably have more important contribution. After recombination was suppressed on the Z chromosome in female, its effective population size becomes smaller than autosomes, and it maybe further reduced due to the variation in male mating success [61]. Genetic drift thus would fix excessive slightly deleterious mutations in synonymous sites, due to Hill-Robertson interference among linked loci. This effect explains the trend of d*S* among evolutionary strata with different ages of becoming Z-linked, of which ‘older’ Z-linked genes tend to be fixed with more slightly deleterious mutations. In addition, we also find the G + C content at the third codon position (GC3) shows a similar trend with that of intron (GCi), between Z/autosomes and different strata (Figure 1D-E) across species (Additional file 1: Figure S5-6). The positive correlation between the G + C content and synonymous substitution rate is consistent with the pattern that has been previously reported in repetitive sequences of chicken [62]. These results together argue against selection is shaping codon usage bias and evolution of synonymous sites in birds, and suggest mutational bias probably plays a dominant role [63].

### Nonsynonymous sites evolve faster in the younger strata of Z chromosome

**Figure 3:**
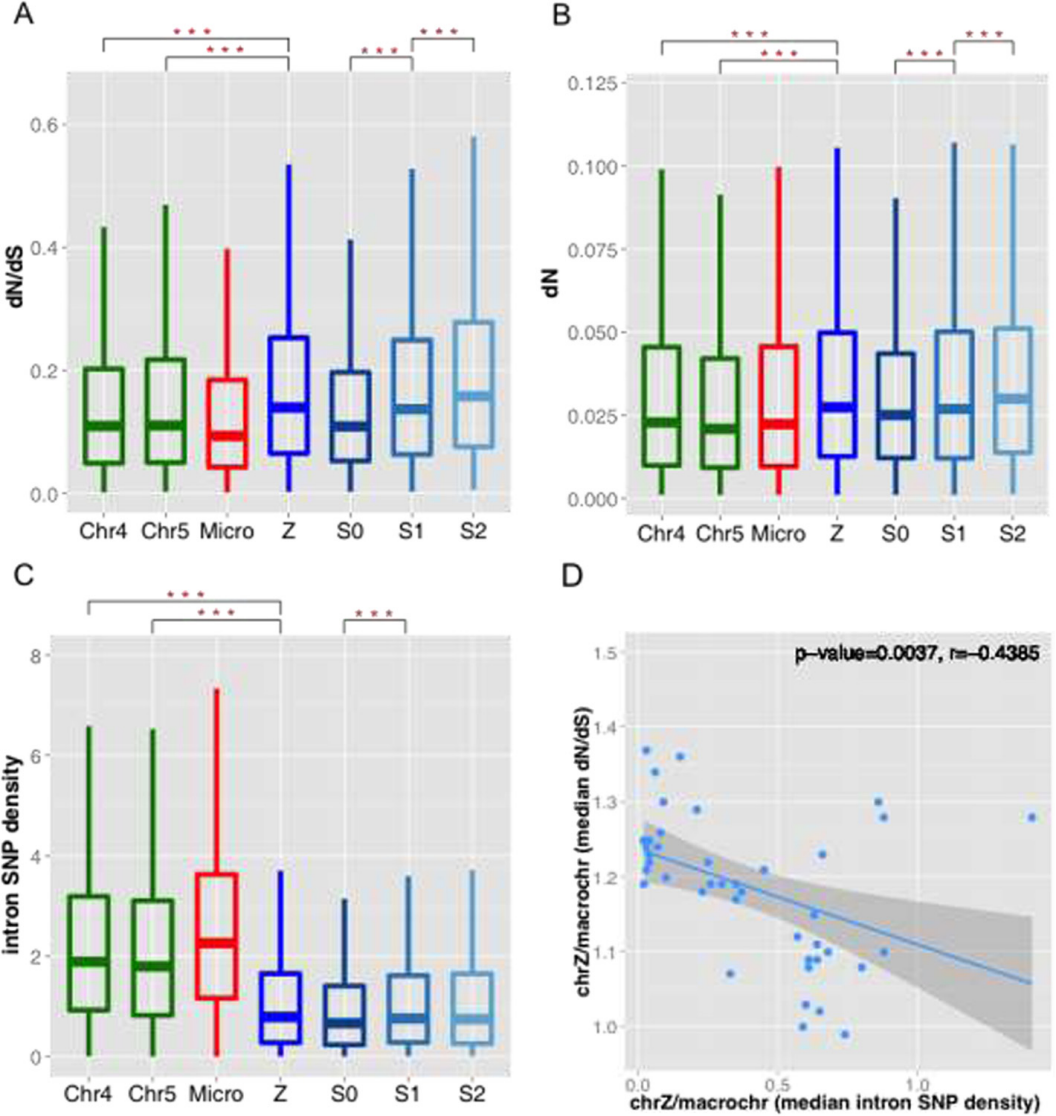
**Evolution patterns of nonsynonymous sites in bird genomes. (A-C)** We show sequence patterns in nonsynonymous sites of coding regions divided in different chromosome sets or different evolutionary strata of Z chromosomes. Chr4 and chr5, which have a similar size and gene number with Z chromosome, are shown, and we also performed the comparison between Z chromosome vs. all macrochromosomes. We show pairs of comparison with significant difference labeled with asterisks. ^***^:*P*-value < 0.001. *d*N: lineage specific nonsynonymous substitution rate. Intron SNP densities were measured in SNP number per kb region. **(D)** For each species, we calculated the ratio of median intron SNP density of Z linked genes vs. macrochromosome linked genes as a proxy for relative effective population size. We also calculated the ratio of median evolutionary rate (*d*N/*d*S) of the two, as a measurement of fast-Z evolution.

On the other hand, we find the ratio (*d*N/*d*S) of nonsynonymous substitution rate over synonymous substitution rate of Z-linked loci is significantly (*P* < 0.05, Wilcoxon test, Figure 3A) higher than that of macrochromosome loci in 34 out of 45 (75.6%) species (Additional file 3: Table S2), showing a pattern of ‘fast-Z’ evolution. This is mainly caused by the elevation of *d*N (Figure 3B) on the Z chromosomes, indicating an excessive accumulation of amino acid changes within the Z-linked genes. In addition, *d*N shows an opposite trend to *d*S among different evolutionary strata, i.e., the younger strata tend to evolve even faster at nonsynonymous sites (Figure 3B, Additional file 1: Figure S7-S8).

Analogous to the ‘fast-X’ evolution patterns reported in *Drosophila* and human, fast-Z evolution may result from a more efficient fixation of recessive mutations which are beneficial to females [15]. This predicts an enrichment of functional categories that are related to the female reproduction process or sex-biased gene expression among genes that are fast-evolving on the Z chromosome. To test this, we compare the Gene Ontology (GO) terms in which candidate genes undergoing adaptive evolution on the Z chromosome and autosomes are enriched. However, we haven’t found any GO terms directly related to sexual reproduction processes (Additional file 4: Table S3) for the Z-linked genes. Instead, macro- or micro-chromosomes are enriched for fast-evolving genes with GO terms like ‘female gonad development’ (GO: 8585), ‘ovulation cycle process’ (GO: 22602) and ‘regulation of reproductive process’ (GO: 2000241) etc. Consistent with the previous analysis [33], sex-biased expression pattern also doesn’t seem to affect the evolutionary rate (measured by *d*N/*d*S ratio) of Z-linked genes. We examine the brain and liver sex-biased gene expression level in both ostrich and chicken (also see below), and study their correlations with evolutionary rates with Z and autosome linked genes in separate (Additional file 1: Figure S9). In contrast to the pattern found on X chromosomes of *Drosophila* [64] and mammals [19], female-biased or non-biased genes do not necessarily evolve significantly faster than male-biased genes on the Z chromosome.

These results are consistent with the previous findings on Z-linked genes of chicken [33,34] and snakes [65], but not silkmoths [66]. They suggest the alternative explanation for the fast-Z evolution, i.e., fixation of slightly deleterious mutations by genetic drift [33-35]. The effective population size of chrZ (*N*_e_z) is three quarters of that of autosomes (*N*_eA_), under the hypothesis of random mating and equal numbers of males and females within population. However, this hypothesis is usually invalid due to the variation in male mating success caused by sexual selection or social mating system [67]. This will result in a much smaller ratio of *N*_ez_/*N*_eA._ As shown by previous theoretical simulation, this ratio has great impact on the degree of fast-X or fast-Z evolution [35]. Indeed, when we compare the neutral polymorphism level approximated by intron SNP densities on the Z chromosome and autosomes, we found the Z chromosomes show a polymorphism level that is significantly (*P* < 0.001, Wilcoxon test) lower than three quarters of that of autosomes (Figure 3C). And the reduction in neutral polymorphism level of Z shows a significant negative correlation (*P* = 0.0037, Pearson’s correlation test) with its increase of lineage-specific evolutionary rate relative to autosomes (Figure 3D), providing direct evidence that the fast-Z evolution of birds is mainly driven by genetic drift. Under this scenario, majority of excessive mutations fixed on the Z chromosome are slightly deleterious. So a larger *d*N or *d*N/*d*S value in younger strata (Figure 3B-C) is probably not caused by recent adaptive evolution. Instead, it may reflect older strata are undergoing stronger purifying selection against slightly deleterious mutations, since they have become hemizygous in female at an earlier time point and exposed recessive deleterious mutations to natural selection for a longer time span. We have recently shown W chromosomes of many bird species degenerate slowly after recombination was suppressed [4]. Therefore, in younger strata like S2, recessive deleterious mutations may still be sheltered on the Z chromosomes by the residual W-linked genes, which probably accounts for a higher *d*N value in the younger strata.

Finally, to test the correlation between male mating success vs. the fast-Z evolution in birds, we study several life history traits that indicate or impact the strength of male sexual selection including residual testis mass, social mating systems, color dichromatism and tail sexual dimorphism [67]. None of these traits show significant correlation with the ratio of evolutionary rate between Z chromosome and autosomes (Additional file 1: Figure S10). This could due to a lack of enough sample species. For example, most studied species are characterized as monogamous species; therefore it is difficult to evaluate the impact of different social mating systems. Also, life history traits can rapidly turn over between species while our measurement of fast-Z evolution is a result of long-term evolution.

### Gene expression become more male-biased over time on the Z chromosome

Z chromosomes are transmitted in a male-biased manner, and may be subjected to selection for dosage compensation, after they suppressed recombination with W and became hemizygous in female. The former predicts more male-biased gene expression on the Z chromosome, which is counteracted by the latter. To examine relative importance of the two, we finally investigate male vs. female expression ratio of Z-linked genes across four species (ostrich, emu, chicken and zebra finch) representing the major groups of avian species over their course of recombination suppression.

**Figure 4:**
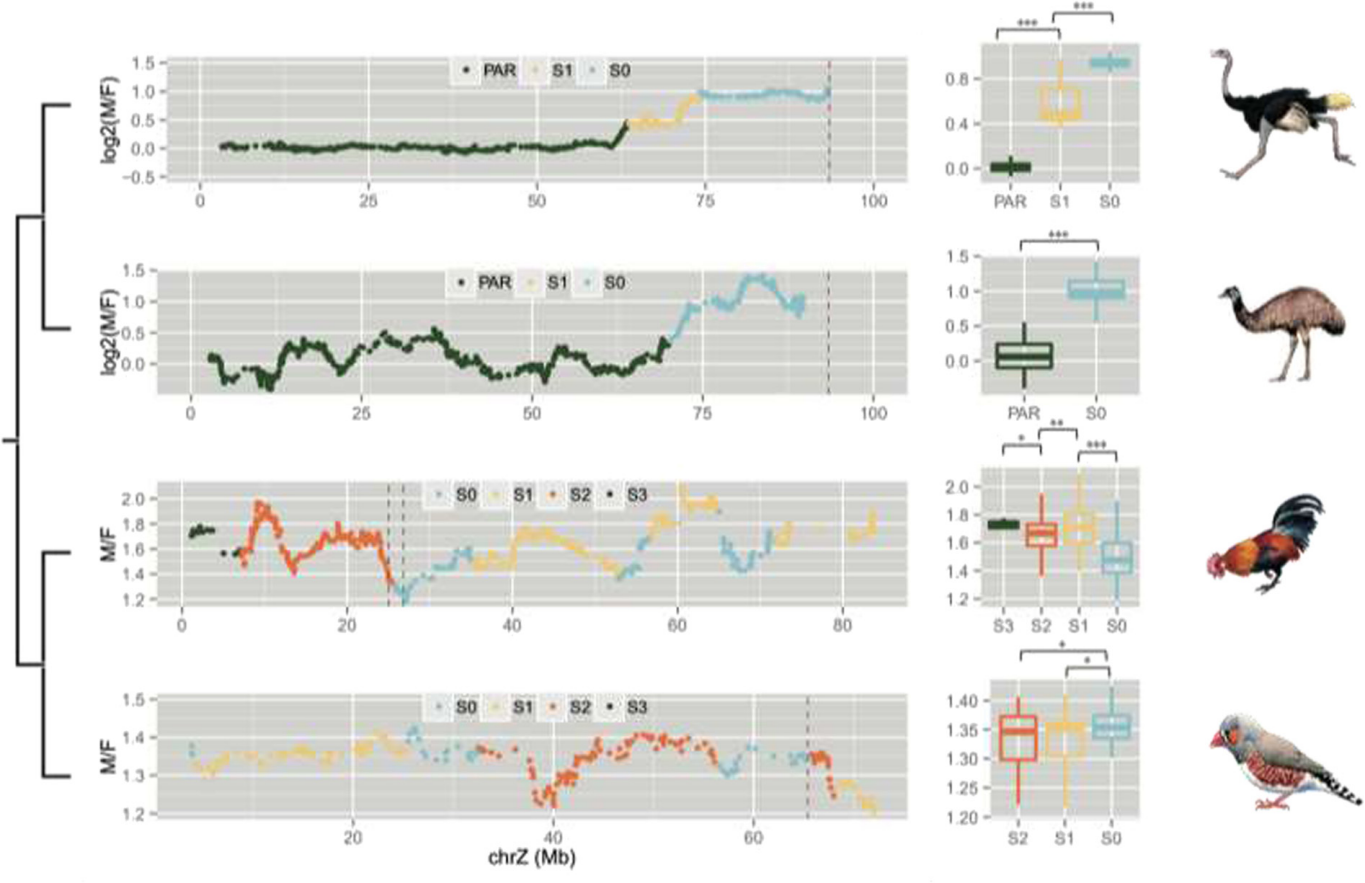
**Z chromosome gene expression become more male-biased over time.** We show male-to-female gene expression ratio along the Z chromosomes of four species that represent the major avian clades. We used the published microarray data of chicken and zebra finch, and RNA-seq data of ostrich and emu. Given the difference of data scale, we present them in log or non-log based ratio. We color-coded the different evolutionary strata, with PAR or S3 in green, S2 in orange, S1 in yellow and S0 in blue. We also marked the putative male-determining genes *DMRT1* with red dotted line in each species. Except for chicken, the other three species show a significantly higher male-biased expression in the older strata, as shown in the boxplots. We marked the non-coding RNA gene *MHM* [28,69] that mediates regional dosage compensation in chicken with green dotted line. Each box is compared to the younger one adjacent to it; the one with significant difference is shown with an asterisk at the bottom. The level of significance: ^*^:*P* < 0.05; ^**^:*P* < 0.01; ^***^:*P* < 0.001.

Intriguingly, we find that, except for chicken, Z-linked genes that are located in the older evolutionary strata and have experienced a longer time of hemizygosity exhibit a significantly higher level of biased expression toward males (Figure 4), compared to others located in the younger strata. In particular, in ostrich and emu, whose sex chromosomes are probably the closest to the ancestral state of avian sex chromosomes among all bird species [4], genes located in the oldest stratum (S0) show a nearly two-fold expression difference between sexes (Figure 4). This Z-linked region encompasses the putative avian male-determining gene *DMRT1* [68], and probably has experienced the first time of recombination inhibition that is shared by all bird species [4]. Therefore, dosage compensation probably has never evolved in response to the emergence of sex determination gene in the ancestor of bird species. In addition, we cannot identify the ortholog of the male hypermethylated (MHM) non-coding RNA gene [28] in charge of the chicken regional dosage compensation in either ostrich genome, or *de novo* assembled transcriptome (data not shown). Extensive intra-chromosomal genomic rearrangements seem to have occurred surrounding chicken *MHM* and *DMRT1* loci after chicken diverged from ostrich and emu based on our genomic analyses (Figure 4, Additional file 1: Figure S11) and previous comparative karyotyping [44], which might have given birth to the *MHM*. These results together suggest the reported regional dosage compensation in chicken [28] is more likely to be recently derived only in chicken or the ancestor of Galloanserae (chicken and duck). Contrary to the previous result [42], we find S0 genes show a lower degree of male-biased expression (male vs. female median expression ratio as 1.5) than other strata, since part of the chicken S0 region defined in this study is close to the center of regional dosage compensation (Figure 4). However, in zebra finch, which doesn’t have this form of dosage compensation [28,69], we did find the S0 genes exhibit the strongest pattern of male-biased expression (Figure 4).

Due to the lack of global dosage compensation, Z chromosomes show a significantly lower expression level relative to other chromosomes (*P* < 0.05, Wilcoxon test, Additional file 1: Figure S12) in female tissues and are generally male-biasedly expressed. However, we have not found a significant difference of overall testis gene expression level between the Z chromosome and autosomes in chicken (*P* = 0.699, Wilcoxon test, Additional file 1: Figure S12), suggesting the male-biased expression of Z chromosome is simply a result of lack of dosage compensation, rather than being driven by sexual antagonistic selection due to its male-biased transmission. This is consistent with the enriched GO results shown above, i.e., there’s no excessive Z-linked male-related genes driven by adaptive evolution. However, this pattern needs to be validated in more bird species without any form of dosage compensation to confirm its generality.

## Conclusions

A complex suite of forces is driving the evolution of sex chromosomes apart from their autosomal ancestors or other autosomes within the same genome [10,16]. One great advantage of studying Z chromosome is it parses the factors of maleness and hemizygosity combined on the X chromosome. We find in this work, most sequence and expression patterns of Z chromosome that we observed across different bird species can be explained by its male-biased transmission, including its higher mutation rate, but not a higher adaptive evolution rate. We find the effective population size of Z chromosome is significantly reduced than that of autosomes under the neutral expectation (Figure 3D), and such a reduction probably results in fixation of excessive slightly deleterious mutations by genetic drift on the Z chromosome, producing a pattern of ‘fast-Z’ evolution across different bird species. By contrast, variation in male mating success that reduces the relative effective population size of Z would instead increase that of X, thus the efficiency of natural selection. This may explain why it is easier to detect adaptive evolution as a cause of ‘fast-X’ evolution [34,35].

We also show the local dosage compensation in chicken [28] may have evolved very recently, and global dosage compensation probably never evolved on the Z chromosome at the common ancestor of birds. Although it is still unclear why female heterogametic systems generally lack global dosage compensation [28], we find regions that lost recombination more recently in birds don’t immediately reduce their gene expression in female to half of the male (Figure 4). This can be attributed to both the slow degeneration of W-linked genes [4] and transcriptional buffering [70] of Z-linked genes, which together might reduce the selective pressure for dosage compensation.

## Methods

### Estimating the degree of male-driven evolution

The sequencing, assembly and annotation of the genomes are described in [38]. We used the orthologous intronic alignments generated by SATé [71] for all sequenced bird species, after removing ambiguous alignment regions [43]. We calculated the pairwise substitution distance between certain species vs. chicken by the baseml program in the PAML package [72]. We used the median value of Z-linked and macrochromosome linked introns’ substitution distance, and the approach of [73] to estimate the *α* value for each species, using the equation *α =* (3Z/A-2)/(4-3Z/A) (‘Z/A’ stands for the divergence ratio of Z-linked vs. autosome linked loci). We further estimated the confidence intervals of *α* by the nonparametric bootstrapping method described in [31], with the bootstrapping process repeated 1000 times.

Life history traits for all the studied bird species used for the regression analyses were collected from the published work. Basal metabolic rate data is from [74]; residual testis size, body mass, clutch size information is from [67,75]; life span and generation time data is originally collected from Encyclopedia of Life website (www.eol.org) then manually curated by Frank Rheindt at National University of Singapore. We used BayesTraits (http://www.evolution.rdg.ac.uk/BayesTraits.html), and the recently resolved phylogenetic tree of the studied 45 species [43] to account for the phylogenetic dependence in our regression analyses [76]. In brief, we first tested whether a specific trait’s evolutionary change is fully dependent on the phylogenetic structure (*λ = 1* implemented in BayesTraits) by maximum likelihood ratio test. Then we used Continuous module of BayesTraits to test the significance of the correlation if certain trait shows the dependence. Otherwise, we performed generalized linear model analysis to test the significance of correlation between multiple trait data vs. *α.*

### Demarcate the evolutionary strata

We divided all Z-linked genes into groups with different ages of becoming Z-linked based on the information of avian evolutionary strata that we inferred recently [4]. In brief, we first inferred the sex of the sequenced bird samples by comparing the sequencing read depth level between Z chromosome vs. autosomes. Those show similar level of depths are recognized as male and confirmed with sample record, and those show reduction in depth on the Z chromosome are recognized as female. We assembled fragments of W-linked sequences for all the species with female sequenced by SOAPdenovo [77], and then aligned and ordered these fragments along the Z chromosomes using chicken or ostrich chromosome sequence as reference. Since suppressed recombination between the avian Z and W chromosomes occurred in a stepwise manner [6,42], each instance of recombination suppression would render the affected region show a distinctive pattern of Z/W pairwise sequence divergence from the nearby regions. We scanned the Z/W sequence divergence with a stepwise window, and determined the boundaries of strata when significant differences of divergence level between adjacent windows were detected. The age of each stratum was further inferred by constructing gene trees using Z and W linked gametolog sequences of multiple species, if available. A gene tree showing the clustering of Z/W gametologs of the same species suggests the residing stratum emerged after the speciation event; a clustering pattern of Z gametologs of different species separated from W gametologs suggests the stratum emerged at the ancestor of the species [78]. The oldest stratum where the earliest recombination suppression between Z/W occurred with the highest Z/W divergence level is termed strata zero (S0), and then S1, S2 etc. They also form a gradient of the time span for which each has become truly Z-linked. For species with completely degraded W chromosomes (e.g., chicken), we inferred their strata information through the orthologous relationship with their related species that has the information.

### Sequence analysis

We hypothesized the preferred codons are generally conserved among different bird species with few exceptions, as reported among Drosophila [79] and plant species [80]. We used CodonW (http://codonw.sourceforge.net/), and the frequency of chicken optimal codon indices [63] to study the codon usage bias of all the other avian species’ genes.

To calculate gene evolutionary rate, we used multiple sequence alignments of the orthologous genes’ coding regions as input derived from [81] for codeml package of PAML. The alignments have been removed for ambiguously aligned regions by GBLOCK [82] and manual inspection. We calculated lineage-specific *d*N, *d*S and *d*N/*d*S ratios under the ‘free-ratio’ (model = 1) model, which assumes a different evolutionary rate along each lineage. We then filtered genes with *d*N or *d*S value higher 2 or lower than 0.001 according to a previous study that characterized the saturation of *d*S in birds [83], before comparing the entire sets of values between Z chromosome and autosomes. To identify the fast-evolving genes, we compared the two alternative models with one assuming all lineages evolve with the same rate (‘one-ratio’ , model = 0), and the other assuming one rate along the focus lineage and one different rate on all the other (background) lineages (‘two-ratio’ , model = 2). Genes evolve significantly faster than the background lineages according to maximum likelihood test are characterized as candidate genes undergoing positive selection. These genes of different species were pooled and finally subjected to GO enrichment analyses based on annotation of their chicken orthologs, by Cytoscape v.2.8 [84], using gene set of the entire chicken genome as background, with hypergeometric test and a significance cutoff of *P* < 0.01.

We aligned the genomic reads against each species genome by bowtie2 [85] and then screened the alignment results by a mapping quality of 20. We used SOAPsnp [86] and snpEff [87] to identify SNP sites within intronic sequences of bird genomes, of which male sample have been sequenced. SNP sites with mapped read depths lower than 4 or higher than 100 were filtered; we also removed those clustered SNP sites whose distance from each other is lower than 5bp.

### Gene expression analysis

We downloaded the RNA-seq reads of both sexes of ostrich, emu, chicken and zebra finch from NCBI SRA database (http://www.ncbi.nlm.nih.gov/sra), which were collected by previous studies [40,41,69,88]. We aligned the reads to each species’ genome by tophat [89] after quality-trimming the reads, and calculated the normalized gene expression level FPKM (Fragments Per Kilobase of transcript per Million mapped reads) by cufflinks [90] for each gene. We annotated and ordered the genes of emu using ostrich chromosomal sequence and genes derived from [4] as reference. Then we scanned the male vs. female expression ratio along the Z chromosome of each species by a five gene non-overlapping window.

We used SOAPdenovo-trans [91] to assemble pooled reads from female ostrich and emu into transcriptomes. Then we searched chicken *MHM* locus’ homolog in ostrich genome or ostrich/emu transcriptome by BLAST (http://blast.ncbi.nlm.nih.gov/Blast.cgi).

## Additional files

Additional file 1: Figure S1. Patters of pairwise intronic substitution distances of 45 bird species. **Figure S2.** The correlation between life history traits and the degree of male-driven evolution. **Figure S3.** Patters of branch-specific synonymous substitution rates of 45 bird species. **Figure S4.** Patters of frequency of optimal codons of 45 bird species. **Figure S5.** Patters of G + C content at the third codon positions of 45 bird species. **Figure S6.** Patterns of G + C content in introns of 45 bird species. **Figure S7.** Patterns of lineage-specific evolutionary rate (*d*N/*d*S) of 45 bird species. **Figure S8.** Patterns of nonsynonymous substitution rates of 45 bird species. **Figure S9.** The association of sex-biased gene expression with gene evolutionary rate. **Figure S10.** The correlation between life history traits vs. fast-Z evolution. **Figure S11.** Extensive intra-chromosomal genomic rearrangements occurred surrounding the chicken *MHM* locus. **Figure S12.** The distribution of gene expression level across different chromosome sets. Additional file 2: Table S1. Degree of male-mutational bias of 44 bird species. Additional file 3: Table S2. Median values of substitution rates of each species and p-values. We show here Wilcoxon test p-values for comparing substitution rates between Z and autosomes or different evolutionary strata. We also present median values and p-values for individual species. Additional file 4: Table S3. Enriched Gene Ontology terms of fast-evolving genes. We show enriched GO terms for candidate genes undergoing positive selection on different chromosome sets. *P*-values of hypergeometric test and descriptions of GO terms were shown.
