# Supplementary material for: Temporal genomic evolution of bird sex chromosomes
Source: BMC Evol Biol. 2014 Dec 12;14:250. doi: 10.1186/s12862-014-0250-8 (PMC4272511; doi:10.1186/s12862-014-0250-8)
Supplement: Additional file 1: Figure S1. — Patters of pairwise intronic substitution distances of 45 bird species. Figure S2. The correlation between life history traits and the degree of male-driven evolution. Figure S3. Patters of branch-specific synonymous substitution rates of 45 bird species. Figure S4. Patters of frequency of optimal codons of 45 bird species. Figure S5. Patters of G + C content at the third codon positions of 45 bird species. Figure S6. Patterns of G + C content in introns of 45 bird species. Figure S7. Patterns of lineage-specific evolutionary rate (dN/dS) of 45 bird species. Figure S8. Patterns of nonsynonymous substitution rates of 45 bird species. Figure S9. The association of sex-biased gene expression with gene evolutionary rate. Figure S10. The correlation between life history traits vs. fast-Z evolution. Figure S11. Extensive intra-chromosomal genomic rearrangements occurred surrounding the chicken MHM locus. Figure S12. The distribution of gene expression level across different chromosome sets. [file 12862_2014_250_MOESM1_ESM.docx]

**Figure S1 Patters of pairwise intronic substitution distances of 45 bird species**

**
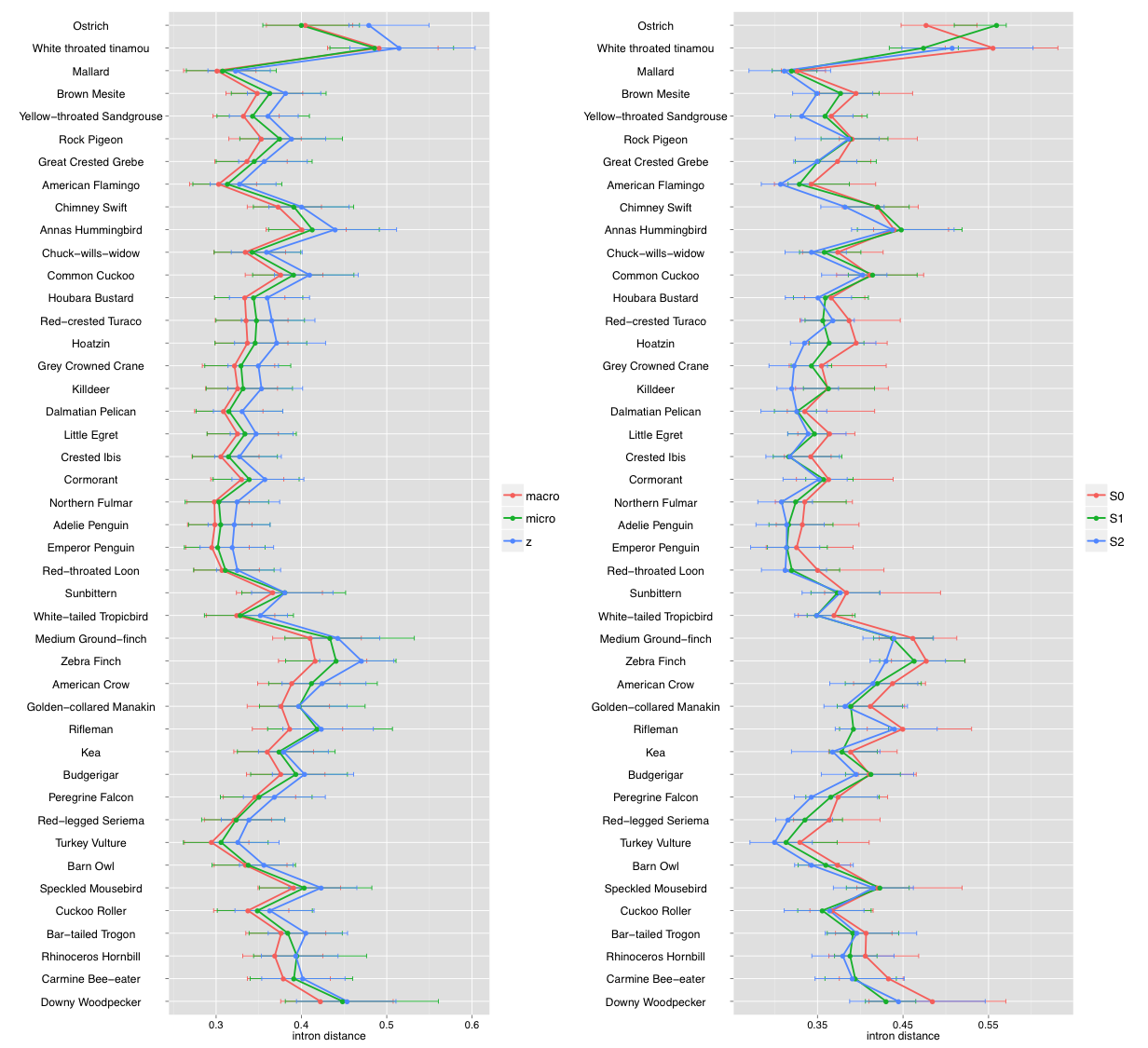
**

**Figure S2. The correlation between life history traits and male-driven evolution**

**
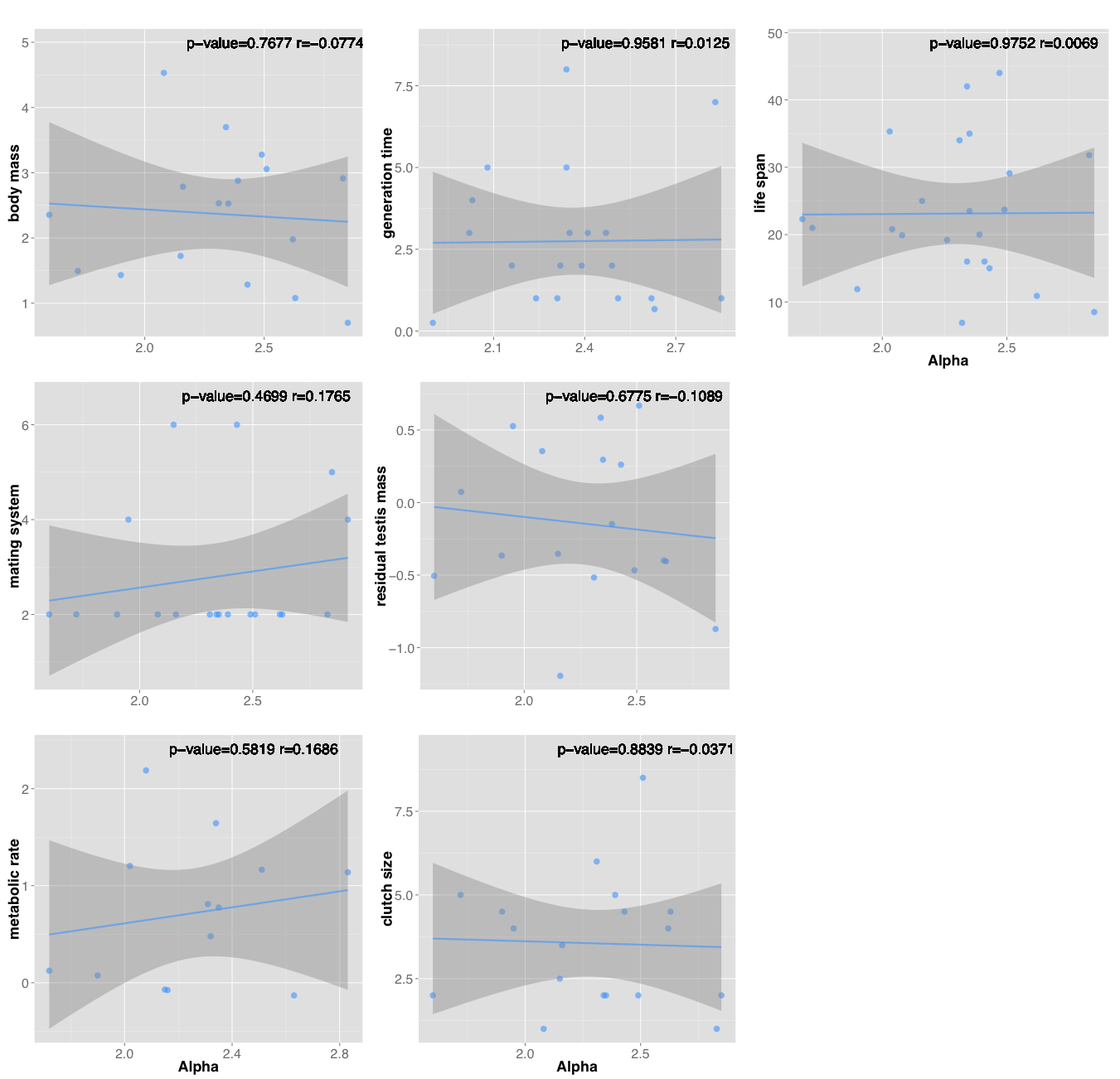
**

**Figure S3. Patters of branch-specific synonymous substitution rates of 45 bird species**

**
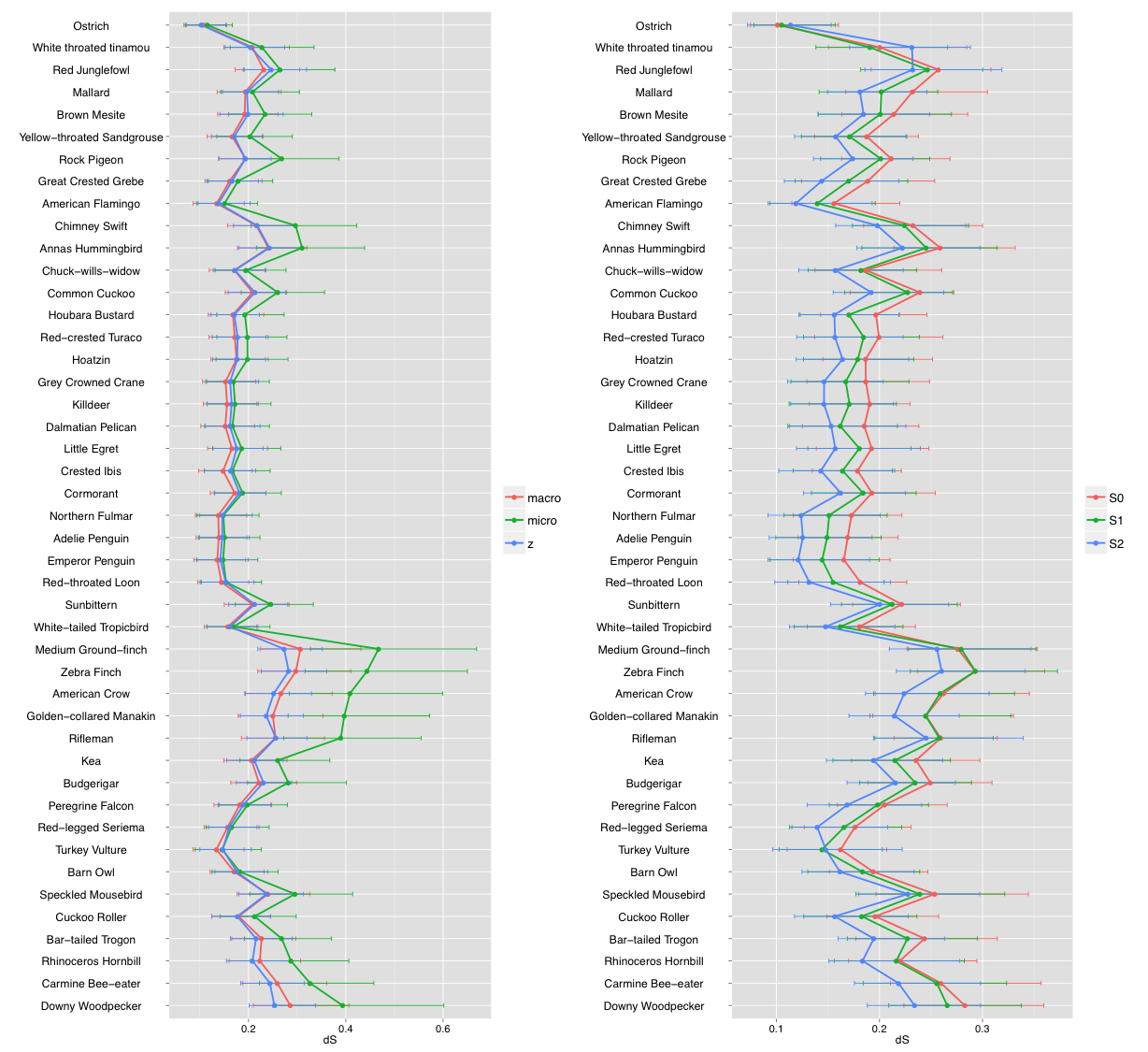
**

**Figure S4. Patters of frequency of optimal codons of 45 bird species**

**
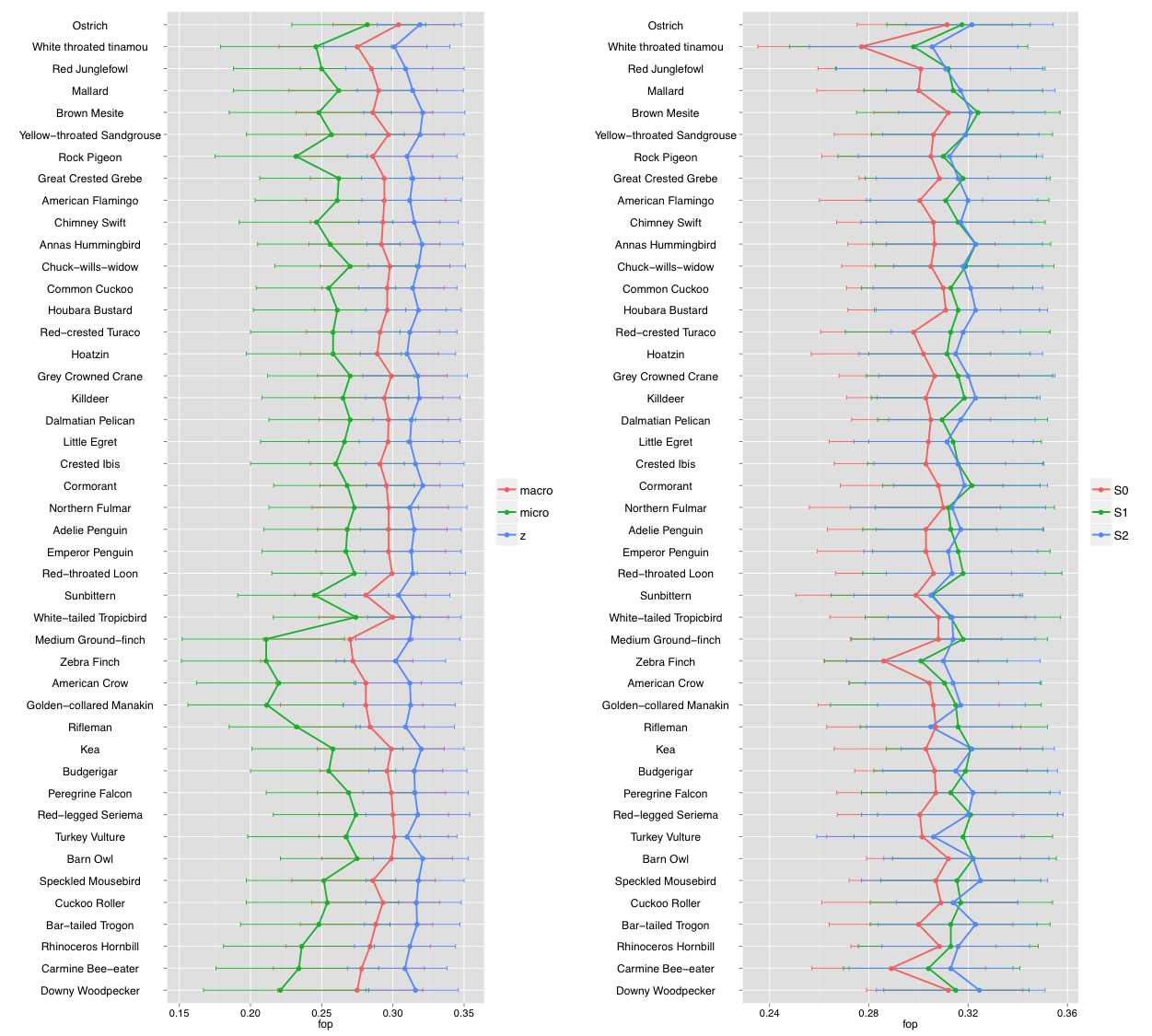
**

**Figure S5. Patters of G+C content in the third codon positions of 45 bird species**

**
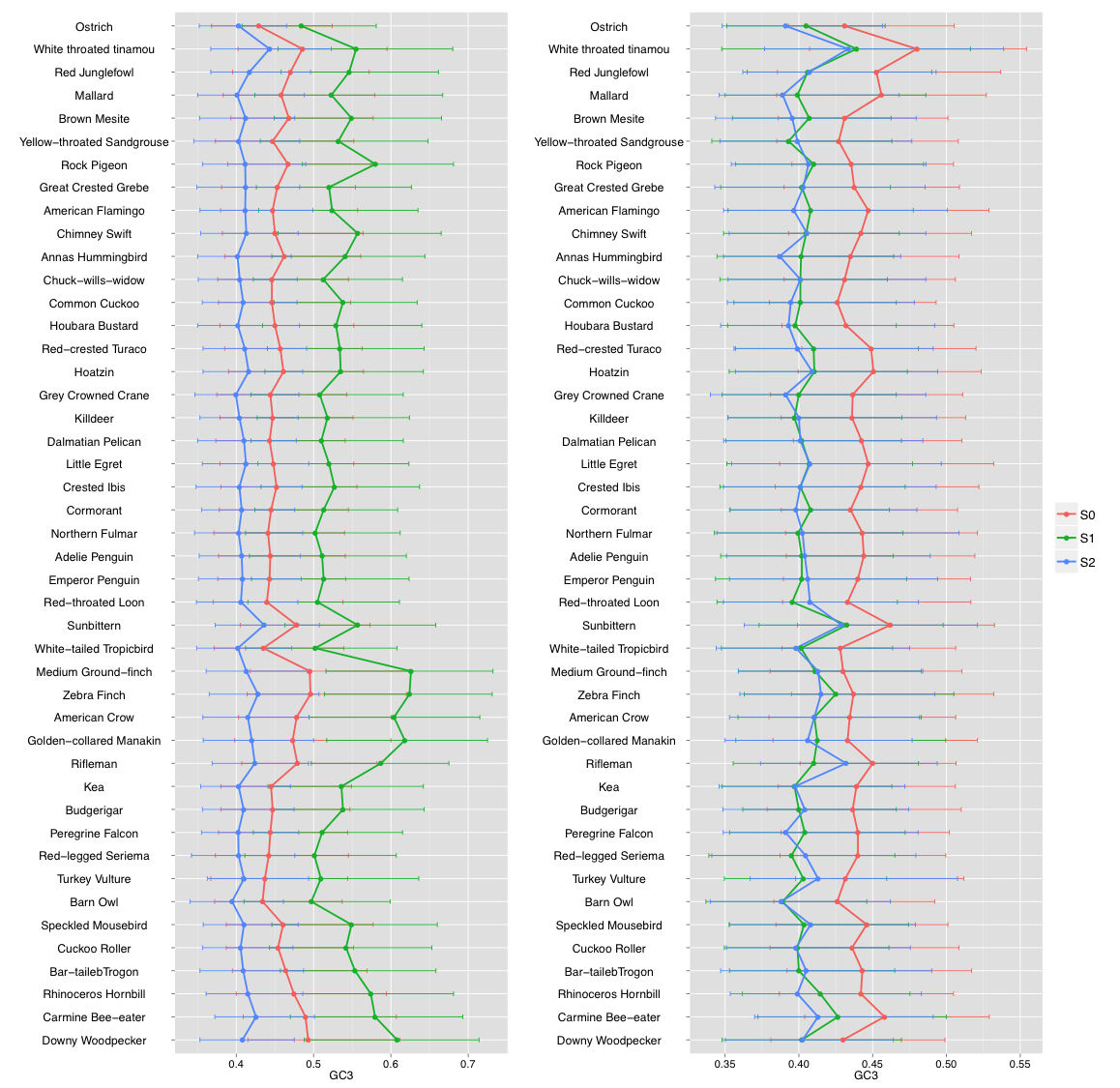
**

**Figure S6. Patterns of G+C content in introns of 45 bird species**

**
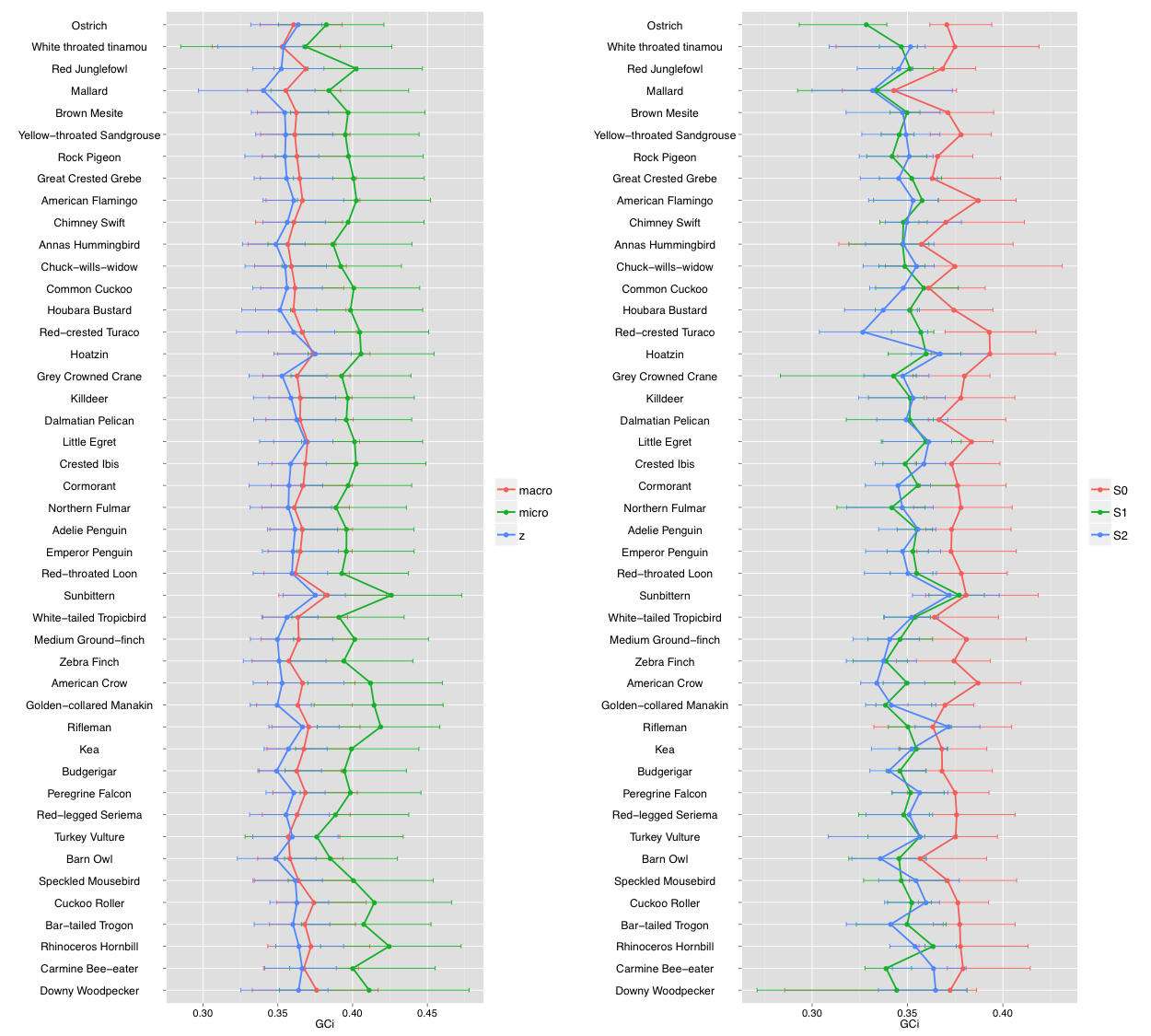
**

**Figure S7. Patterns of lineage-specific evolutionary rate (*d*N/*d*S) of 45 bird species**

**
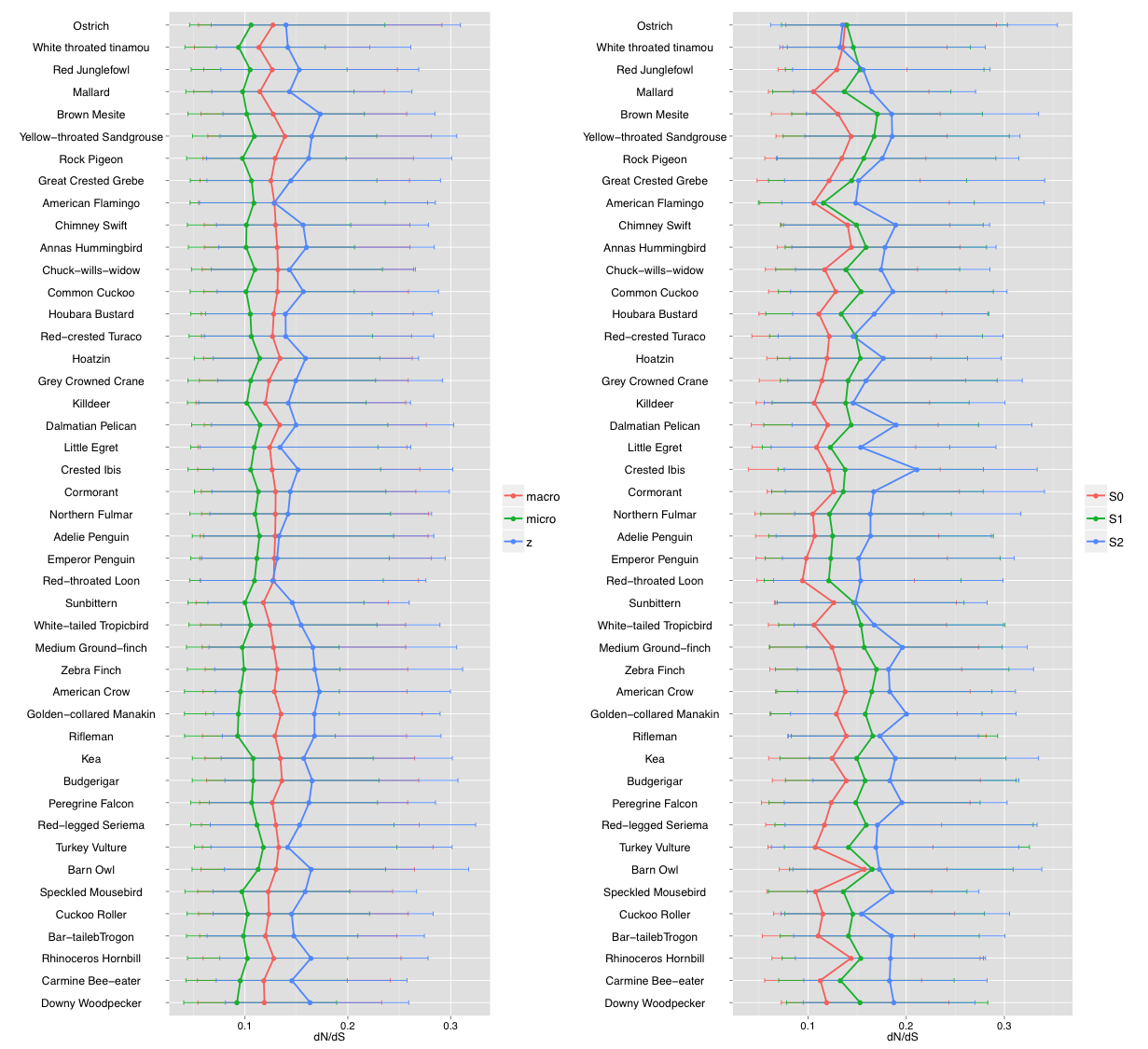
**

**Figure S8. Patterns of nonsynonymous substitution rates of 45 bird species**

**
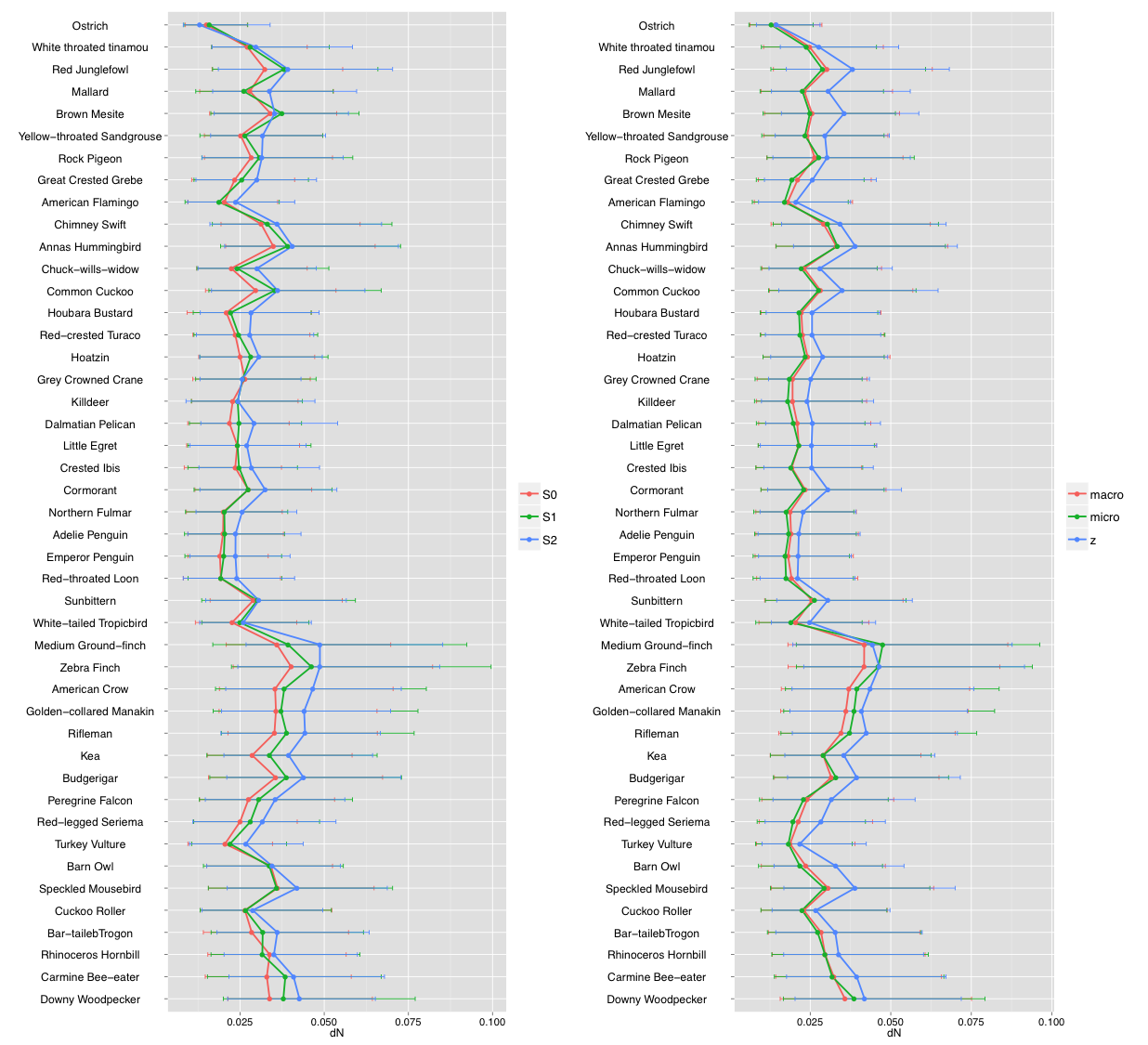
**

**Figure S9 The association of sex-biased gene expression with gene evolutionary rate**

**
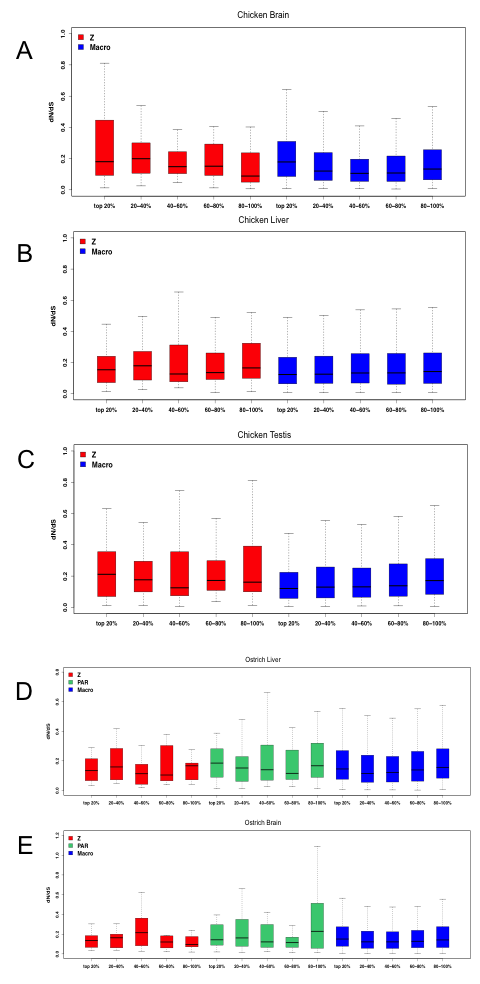
**

**Figure S10 The correlation between life history traits vs. fast-Z evolution**

**
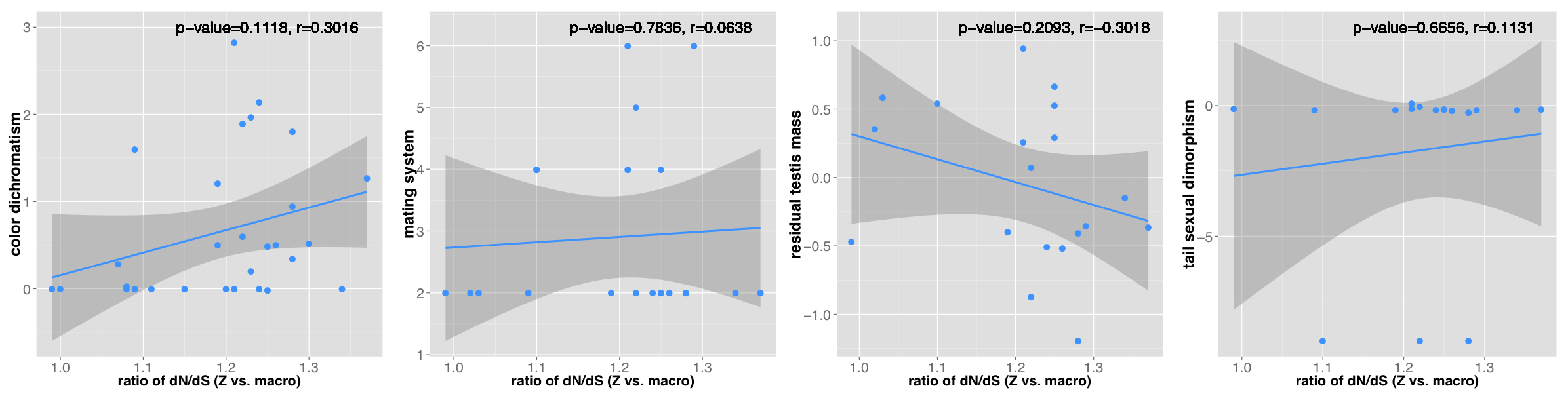
**

**Figure S11. Extensive intra-chromosomal genomic rearrangements occurred surrounding the chicken *MHM* locus**

**
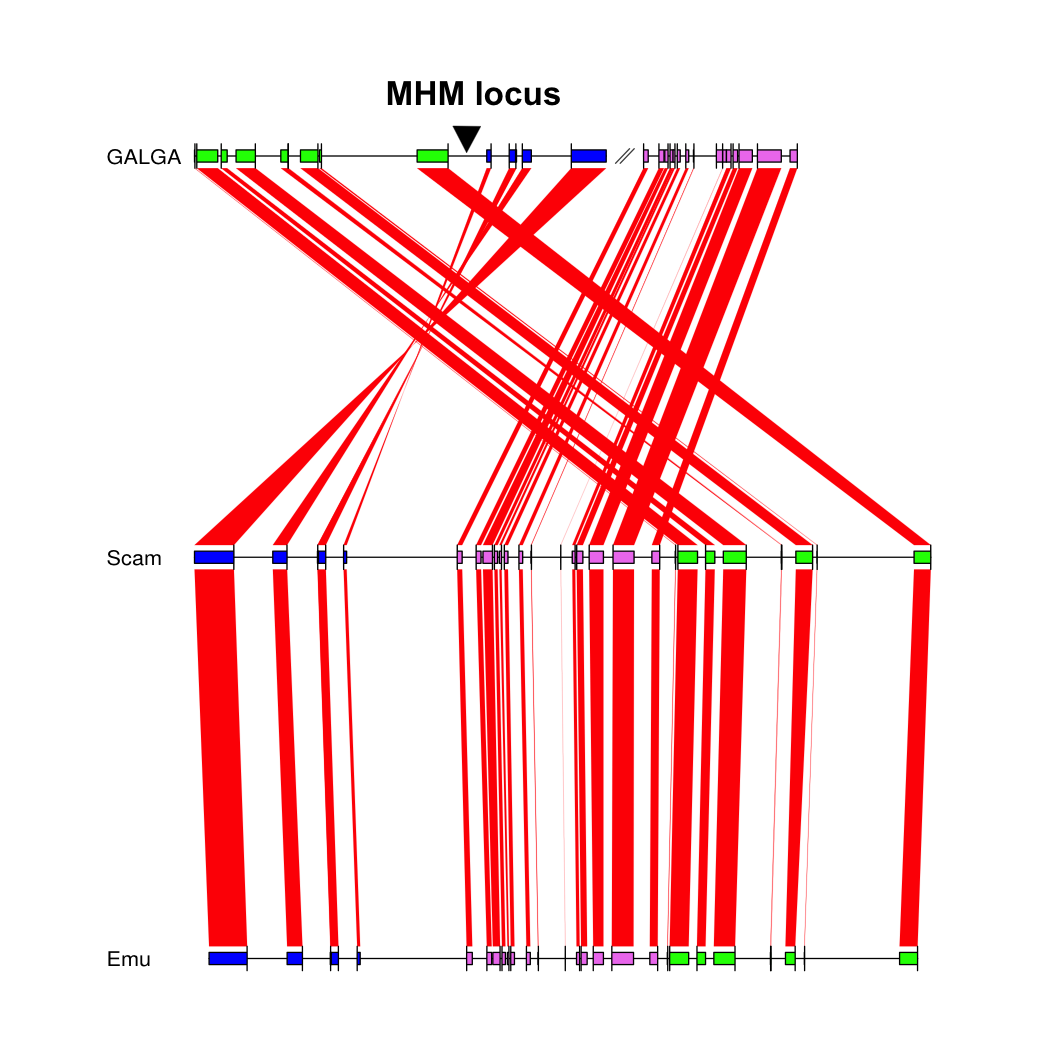
**

**Figure S12. The distribution of gene expression level across different chromosome sets**

**
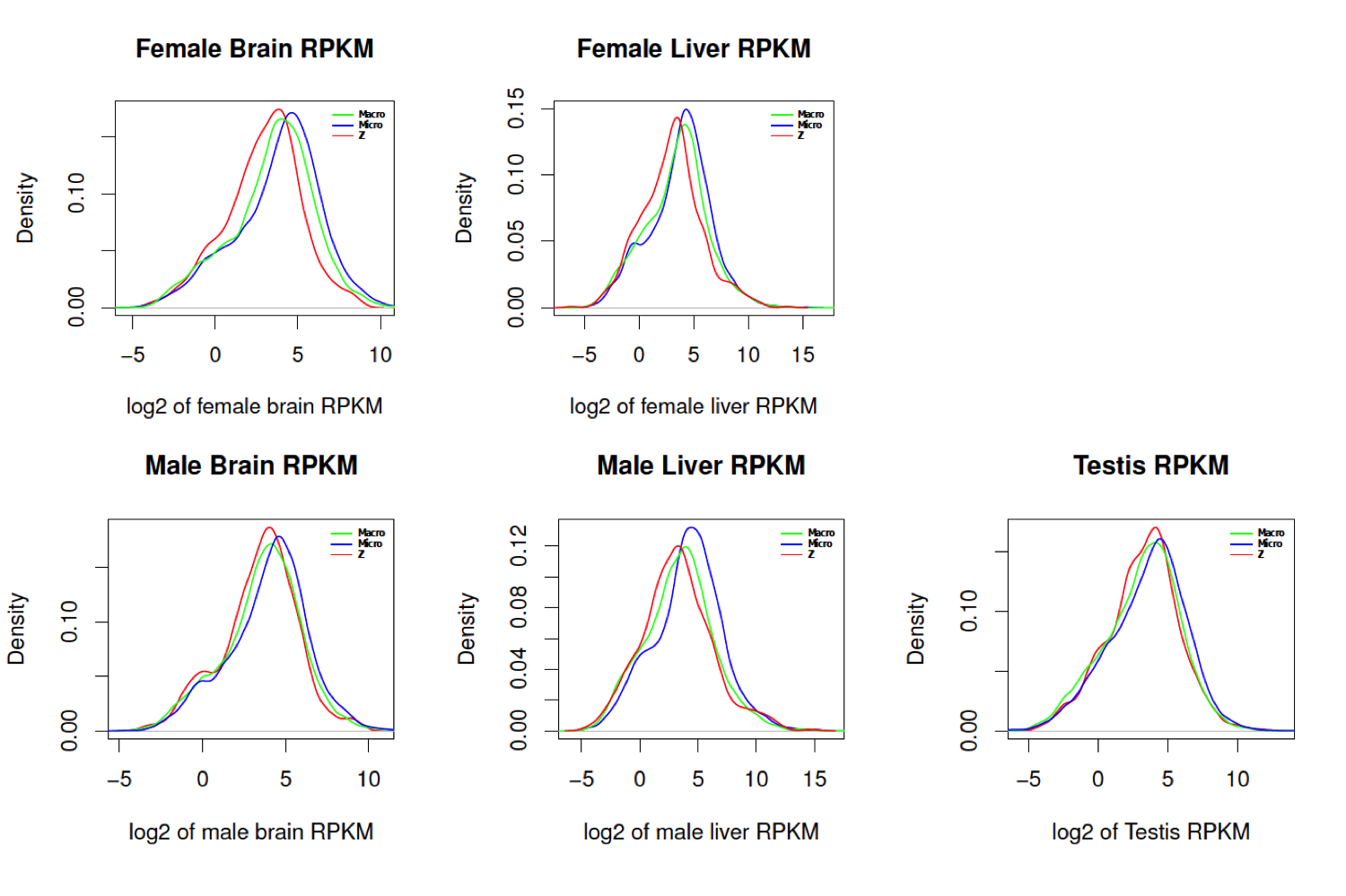
**
